# Evaluation of a Ten-Year Team-Based Collaborative Capacity-Building Program for Pediatric Cardiac Surgery in Uzbekistan: Lessons and Implications

**DOI:** 10.5334/aogh.2883

**Published:** 2020-08-25

**Authors:** Seungheon Han, Sugy Choi, Jongho Heo, Jayoung Park, Woong-Han Kim

**Affiliations:** 1 Institute of International Affairs, Seoul National University, Seoul, KR; 2 Department of Health Law, Policy & Management, Boston University School of Public Health, Boston, Massachusetts, US; 3 National Assembly Futures Institute, Seoul, KR; 4 Program in Global Surgery and Implementation Science, JW LEE Center for Global Medicine, Seoul National University College of Medicine, Seoul, KR; 5 Department of Thoracic and Cardiovascular Surgery, Seoul National University College of Medicine, Seoul, KR; 6 Department of Thoracic and Cardiovascular Surgery, Seoul National University Children’s Hospital, Seoul, KR

## Abstract

**Background:**

Most children who have congenital heart disease in low- and middle-income countries (LMICs), including Uzbekistan, do not receive adequate and timely pediatric cardiac surgical care. To strengthen the surgical capacity of a local pediatric cardiac surgery team in Tashkent, Uzbekistan, the JW LEE Center for Global Medicine at Seoul National University College of Medicine has developed a team-based training program and has been collaboratively conducting surgeries and care in order to transfer on-site knowledge and skills from 2009 to 2019.

**Objectives:**

To evaluate the long-term effects of the collaborative program on the cardiac surgical capacity of medical staff (teamwork, surgical complexity, and patients’ pre-surgical weights) as well as changes in the lives of the patients and their families. To derive lessons and challenges for other pediatric cardiac surgical programs in LMICs.

**Methods:**

To assess the effects of this ten-year long program, a mixed-methods design was developed to examine the trend of surgical complexity measured by Risk Adjustment for Congenital Heart Surgery 1 score (RACHS-1) and patients’ pre-surgical weights via medical record review (surgical cases: n = 107) during the decade. Qualitative data was analyzed from in-depth interviews (n = 31) with Uzbek and Korean medical staff (n = 10; n = 4) and caregivers (n = 17).

**Findings:**

During the decade, the average RACHS-1 of the cases increased from 1.9 in 2010 to 2.78 in 2019. The average weight of patients decreased by 2.8 kg from 13 kg to 10.2 kg during the decade. Qualitative findings show that the surgical capacity, as well as attitudes toward patients and colleagues of the Uzbek medical staff, improved through the effective collaboration between the Uzbek and Korean teams. Changes in the lives of patients and their families were also found following successful surgery.

**Conclusions:**

Team-based training of the workforce in Uzbekistan was effective in improving the surgical skills, teamwork, and attitudes of medical staff, in addition, a positive impact on the life of patients and their families was demonstrated. It can be an effective solution to facilitate improvements in pediatric cardiovascular disease in LMICs if training is sustained over a long period.

## Introduction

Cardiovascular disease is the major cause of mortality around the world [1]. In particular, congenital heart disease, the most common congenital malformation, exhibits negative and long-lasting health and socioeconomic consequences for individuals and society. Most children who have congenital heart disease in low- and middle-income countries (LMICs) do not receive adequate care [2], and about 70% of the congenital heart disease cases in babies require medical or surgical care in their first year [3]. However, few LMICs can provide adequate and timely pediatric cardiac surgical care. It is estimated that approximately 58% of the congenital heart disease burden can be averted if surgical practices from high-income countries are applied to health care settings in LMICs [4]. Strengthening the surgical workforce is one of the ways to improve surgical care in LMICs [5,6]. Training the workforce in pediatric cardiac surgery remains understudied despite its potential impact in LMICs.

Collaboration between a high-income country (HIC) team and an LMIC team has been emphasized as an effective method for successfully training the health care workforce in LMICs [2,7,8,9,10,11]. The JW LEE Center for Global Medicine (CGM) at Seoul National University College of Medicine has developed a team-based training program to build up the surgical capacity of a local pediatric cardiac surgery team in Tashkent, Uzbekistan from 2009 to 2019. CGM transferred their knowledge and skills using the team-based training approach, which emphasized that surgical professionals in LMIC ought to build a team to provide effective surgical care. This methodology has been documented to effectively transfer knowledge among professionals as well as increase positive surgical outcomes by facilitating communication and empathy between team members [12,13,14,15]. A team is generally defined as two or more individuals working together to achieve common goals through sharing common targets, information, and resources based on mutual trust [14,16]. Since the inherent characteristics of healthcare are interdisciplinary, physicians, nurses, and other professionals from different specialties should work together in a team [14]. A considerable amount of literature confirms the effectiveness of this team-based approach among professionals as a critical contributory factor for successful surgical outcomes [13,14,15] and workforce training [2,7,8,9].

The objective of this study was to assess the effects of this program throughout the ten-year period in Uzbekistan. To fully understand and strengthen the research findings [17,18,19,20,21,22], the current study utilized a mixed-methods approach that combined quantitative and qualitative data to evaluate the program’s effects from diverse aspects. Our study examined aspects which included: 1) Changes in teamwork and surgical skills in terms of the complexity of performed pediatric surgeries among surgical staff, and 2) changes in lives of patients and their families. From the findings, we inferred lessons and challenges for the next decade of the ongoing program and other pediatric cardiac programs in LMICs.

## Methods

### Program description

**Figure 1:**
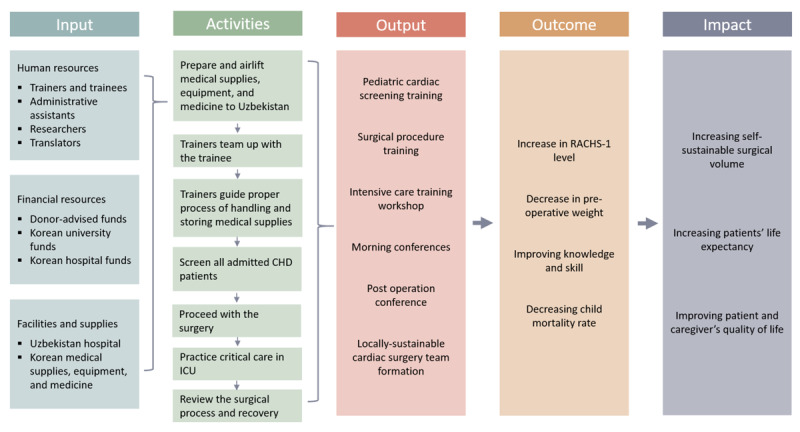
A project design matrix for the on-site cardiac surgical capacity-building program in Uzbekistan.

From 2009 to 2019, an assembled team of Korean cardiac surgeons, pediatric cardiologists, anesthesiologists, perfusionists, intensive care unit (ICU) nurses, administrative assistants, and researchers visited Uzbekistan annually in order to improve the knowledge and skills of pediatric cardiac surgery delivery and patient care in a team, using didactics, discussions, and hands-on training [23]. The Korean team and the Uzbek team engaged in all processes of diagnosis, surgical treatment, and pediatric intensive care. During morning and post-operation conferences, all gathered to recapitulate the surgical procedure of children who underwent open-heart surgery and to discuss the optimization of patient conditions. Didactics were provided according to the Uzbek team’s needs. Unlike other mission teams from other HICs, this particular pediatric cardiac surgical capacity-building program was implemented with several principles: to recruit sicker or more complex patients that the Uzbek medical staff found difficult to operate on; to provide the Uzbek team opportunities to conduct complex surgical procedures collaboratively with the Korean team, or independently but under the supervision of the Korean team (depending on the complexity and familiarity of the cases); to support and emphasize local team-building; and to transfer knowledge and skills through team-based activities between the Korean and Uzbek teams. By virtue of the program’s team-based training approach, the local surgery team would gain the ability to conduct self-sustainable open-heart surgery, ultimately improving access to cardiac care for children with congenital heart disease. The details have been published elsewhere [23]. The design, processes, and outcomes are expressed in a project design matrix for the on-site cardiac surgical capacity-building program in Uzbekistan (Figure 1).

### Data collection and measures

Both quantitative and qualitative data were collected for the duration of the training program from 2010 to 2019. Chosen outcome indicators were teamwork and surgical skills of the medical staff, as our focus was on building teamwork in the local surgical team to increase surgical skills and survival through the collaborative program. Additionally, we checked outcomes from the patients’ perspectives including changes in patients’ health and family life.

First, to quantify the pediatric surgical capacities of the local team, quantitative data to measure surgical complexity during the program were collected via medical record review from 2010 to 2019 (surgical cases: n = 107). Measures included patient pre-operative weight (kg), diagnosis classification, and surgical procedure. Surgical procedures were grouped into six risk categories in the order of increasing mortality risk within a pediatric population using the Risk Adjustment for Congenital Heart Surgery 1 score (RACHS-1) classification [24,25]. A greater number of categories in the RACHS-1 represents progressively more difficult surgical cases. RACHS-1 classification uses surgical procedures to predict the risk associated with the operation and the measure has been used in the context of developing countries [26,27,28]. Two cardiac surgeons independently coded the procedures using the RACHS-1 classification and agreed on the final assignment.

Qualitative data were collected via individual in-depth interviews. In total, 31 interviews were conducted with the Uzbek medical staff (n = 10), caregivers whose child had heart surgery (n = 17), and the Korean medical staff (n = 4), using purposive sampling for maximum variation in profession and length of participation in the surgical program (Table 1). For example, to collect enough information on the process and outcomes of the program, the Uzbek and Korean medical staff were selected as interviewees based on whether they participated in the program and worked together with the Korean staff during 2009–2019. Ranging from one to ten years, all medical staff have participated in the program. We also considered various professions to collect diverse perspectives on the program. Although we did not include the entire medical staff due to time limitations, we made an effort to collect quality data by probing the underlying meanings of their statements. Three interview sessions were conducted; from June 11–16, 2016; from May 13–18, 2017; and on Feb 21, 2020. Bilingual interviewers (Uzbek-Korean) used semi-structured guides to conduct interviews. All interviews were conducted after receiving consent for participation.

**Table 1:** Characteristics of interview participants (N = 31).

Interview groups	Year of interview	Interview Participants						

		Cardiac Surgeon	Perfusionist	Pediatric Cardiologist	Anesthesiologist	ICU Nurse	Ward Nurse	Scrub Nurse

Uzbekistan medical staff (N = 10)	2016	2	1	–	–	–	2	–
	2017	2	–	–	1	1	–	1
	2020	–	–	–	–	–	–	–
Korean medical staff (N = 4)	2016	–	–	–	–	–	–	–
	2017	1	–	1	–	1	–	–
	2020	–	–	–	–	1	–	–
Caregivers (N = 17)	2016							14
	2017							3
	2020							–

Unit: Number of people; ICU: intensive care unit.

We generated different in-depth interview questions according to the interviewee groups. For the Uzbek and Korean medical staff, interviews were conducted to explore the role of the training program, the results of the program, and challenges for developing pediatric medical capacities in Uzbekistan. For caregivers, interviews were conducted to explore a detailed history of the child’s health issues, child’s health and daily life before and after surgery, and life changes of caregivers and other family members before and after surgery. Interviews were conducted in a private room at the hospital, and each interview lasted about 30 minutes on average. All interviews were audio-recorded and transcribed verbatim in Uzbek by a professional transcriber and then translated into Korean.

### Data analysis

For quantitative data analysis, we presented descriptive statistics of the diagnosis classification. To show increases in pediatric surgical capacity within a decade, average outcome values of patients’ RACHS-1 score, pre-operative weight (kg), weight by year, and linear prediction lines were presented. We depicted the linear regression coefficients, 95% confidence intervals, and p-values in the figures (* indicates p < 0.1, ** p < 0.05, and *** p < 0.001). STATA MP 15 (StataCorp, College Station, TX) was used to conduct all quantitative analyses.

For qualitative data analysis, the thematic analyses of qualitative data involved a collaborative process [29]. We used open coding of transcripts to identify key words, phrases, and statements. All researchers read each transcript and identified notable words, phrases, and patterns from the participants’ responses. Finally, responses were categorized into themes related to the research topics of each interviewee group. All quotes are extracted from individual interviews and names are not shown to protect confidentiality.

### Ethical approval and consent to participate

The study protocol was approved by institutional review boards at Seoul National University College of Medicine (IRB 1908-122-1056). Study participation was voluntary and written informed consent in the local language (Uzbek or Korean) was obtained from all participants. Surveyors and interviewers trained in ethical matters collected all data.

## Results

### Quantitative findings: increasing surgical complexity and decreasing pre-operative weight

A total of 107 patients received cardiac surgery during the training program from 2010 to 2019. All patients survived to discharge. Table 2 shows operation characteristics during the training when the Korean team was present.

**Table 2:** Annual patient operation records during the training, 2010–2019.

	2010	2011	2012	2013	2014	2015	2016	2017	2018	2019	Total

**N**	10	11	11	13	12	11	10	10	10	9	107
**Gender, N**											
Female	6	8	4	6	2	7	6	2	5	4	50
Male	4	3	7	7	10	4	4	8	5	5	57
**Age, months**											
Mean	35.9	46.1	35.8	30.2	31.1	16.3	26.3	12.3	42.2	22.3	30.0
(SD)	(30.2)	(38.3)	(40.5)	(22.0)	(37.1)	(31.7)	(27.4)	(11.1)	(41.0)	(25.3)	(32.1)
**Weight, kg**											
Mean	13.0	13.9	11.8	10.3	11.2	6.7	9.1	6.2	12.1	10.2	10.5
(SD)	(8.5)	(5.1)	(7.4)	(4.3)	(5.7)	(5.6)	(4.8)	(3.2)	(8.2)	(6.3)	(6.3)
**Risk Category, RACHS-1**											
Mean	1.9	2	2.4	2.2	2.4	2.7	2.5	2.6	3	2.8	2.4
(SD)	(0.3)	(0.5)	(0.8)	(0.6)	(0.8)	(1.0)	(0.9)	(0.8)	(1.1)	(0.8)	(0.8)

Trainers and trainees mostly performed ventricular septal defect (VSD) (18.7%), Tetralogy of Fallot (TOF) (15.9%), single ventricle (15.0%), and total anomalous pulmonary venous return (TAPVR) cases (13.1%). All cases were placed into categories below 5 (Table 3).

**Table 3:** Diagnosis Classification, 2010–2019.

Diagnosis Classification	N (%) *Total = 107*

Ventricular septal defect (VSD)	20 (18.7)
Tetralogy of Fallot (TOF)	17 (15.9)
Single Ventricle	16 (15.0)
Total anomalous pulmonary venous return (TAPVR)	14 (13.1)
Coarctation of the aorta (CoA)	5 (4.7)
Transposition of the great arteries (TGA), VSD	5 (4.7)
CoA-VSD	3 (2.8)
Ebstein’s anomaly	3 (2.8)
Atrial septal defect (ASD)	2 (1.9)
Complete atrioventricular septal defect (AVSD)	2 (1.9)
Interrupted aortic arch (IAA)	2 (1.9)
Pulmonary atresia with an intact ventricular septum (PA-IVS)	2 (1.9)
Pulmonary atresia with ventricular septal defect (PA-VSD)	2 (1.9)
Complete AVSD with TOF	1 (0.9)
Complete TGA with IVS	1 (0.9)
Complete TGA with VSD	1 (0.9)
Double outlet right ventricle (DORV) with PS (Fallot type)	1 (0.9)
Intracardiac mass	1 (0.9)
Partial anomalous pulmonary venous return (PAPVR)	1 (0.9)
Posterior interventricular artery (PDA)	1 (0.9)
Partial AVSD	1 (0.9)
Pulmonary atresia with VSD	1 (0.9)
Tricuspid Atresia (TA)	1 (0.9)
TAPVR-ASD	1 (0.9)
TOF-PA	1 (0.9)
VSD-ASD	1 (0.9)
VSD-PS	1 (0.9)

**Figure 2:**
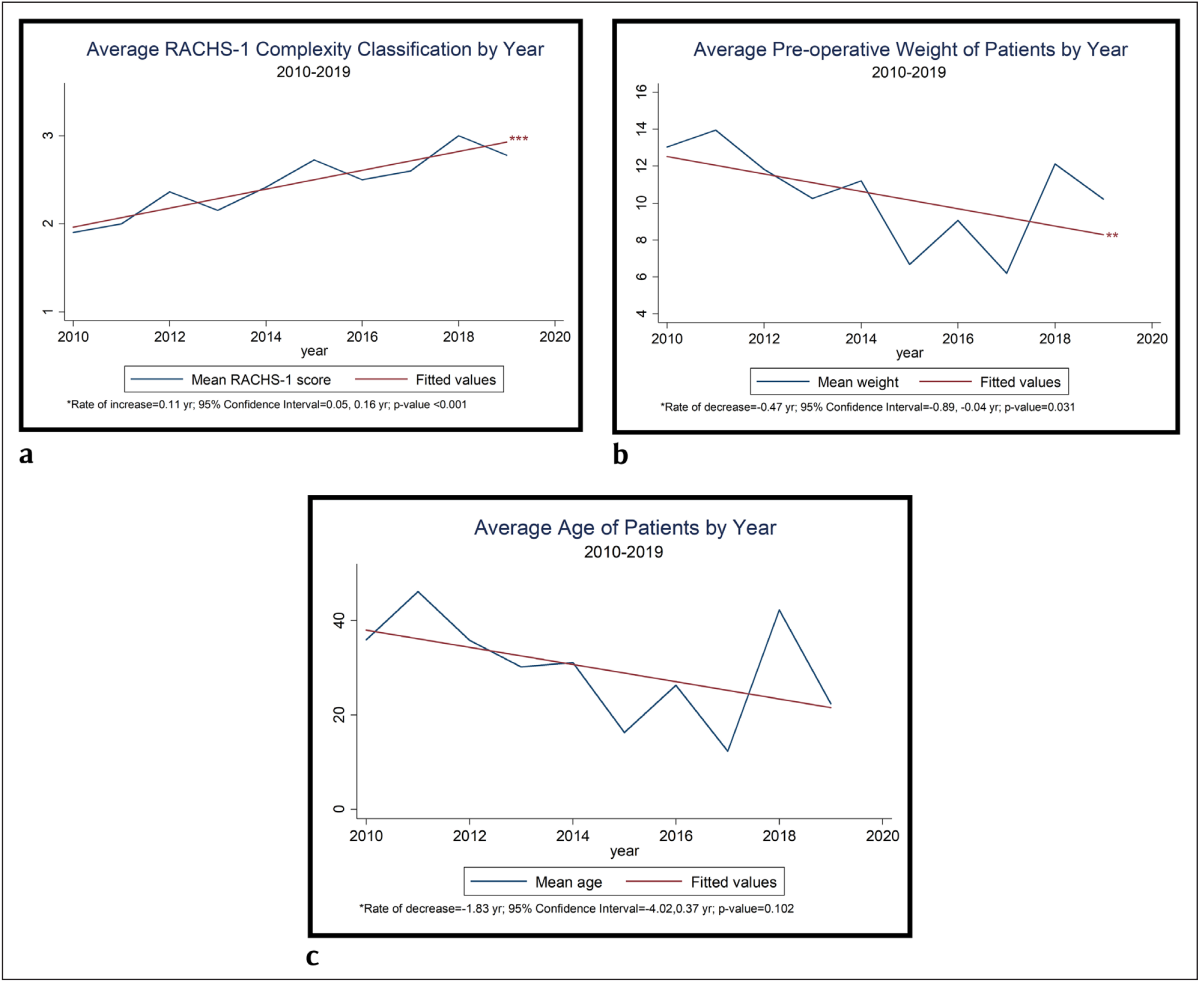
Surgery data and patient characteristics.

Figure 2a shows the unadjusted trends in surgical complexity (RACHS-1) of the cases from 2010 to 2019. On average, the RACHS-1 score increased from 1.9 in 2010 to 2.78 in 2019, showing a gradually increasing trend, and was statistically significant at p < 0.001. Starting in 2018, more than half of the cases exhibited higher severity, where they were assigned 3 or 4 (2018 = 70.0%, 2019 = 55.6%).

Figure 2b shows the unadjusted pre-operative weights of the patients. The average weight of patients decreased by 2.8 kg from 2010 to 2019, thus showing a gradual decrease trend with yearly fluctuation (p = 0.031). The lowest weight was 2.6 kg for a patient who had surgery in 2015. The lowest average patient weight was 6.2 kg, that was achieved in 2017.

Figure 2c shows the unadjusted trends in patient age from 2010 to 2019. The average age of patients (in months) decreased from 35.9 months to 22.3 months (p = 0.102).

## Qualitative findings

### Theme 1: Effective surgical capacity-building with collaborative team-based training

The development of pediatric cardiac surgery in Uzbekistan over the past decade has been supported by effective teamwork between the Korean and Uzbek medical staff. Most Uzbek medical staff emphasized that they could develop their own specialized medical skills and competencies through the Korean ‘whole team’ approach which included major departments such as cardiac surgery, cardiology, anesthesiology, perfusion, ICU nursing, and scrub nursing. According to interviews, other foreign countries had dispatched only one or two surgeons who returned to their home country after operating. In this circumstance, it was difficult for the Uzbek medical staff to learn and develop the expertise they needed.

The other overseas teams came and did not speak to us. However, the Korean team already formed their team before coming here, so it was different from other overseas cases. Each member spoke to its respective counterpart. For example, the anesthesiologists spoke to the anesthesiologists, the internal medicine doctors talked to the internal medicine doctors, and the nurses talked to the nurses. Each Uzbek medical staffer spoke to the Korean medical staffer according to their own department. In doing so, the whole (Uzbek) team developed together. (Uzbek medical staff 5, 2016)

Only two or three people came from the teams from XX, XX, and XX University, and only the surgeon came to do the surgery. But when they did that, the results were not good, and we did not learn much from each other. However, the Korean team came with a team of nurses, surgeons, anesthesiologists, and so on. The result was good and there were many things to learn. The greatest advantage was working together with the team based on roles. (Uzbek medical staff 4, 2016)

Previously, only doctors could calculate an amount of medicine, but nurses were able to do it themselves after training by the Korean team. The Korean nurses taught us how to weigh a newborn baby. Doctors only inform patients about their medications, but now, nurses can do it. It is important for nurses to learn from nurses. (Uzbek medical staff 3, 2017)

### Theme 2: Medical staff’s change in perception and behavior towards patients and colleagues

In addition to the surgical skills that the Korean medical staff directly transferred through teamwork, the perception and behavior of the local medical staff also positively changed in various aspects, including work ethic, responsible attitude toward dealing with colleagues and patients, and perception of teamwork.

As the most noticeable change, the local medical staff were motivated to work harder with sincerity due to the Korean staff working respectably among them.

The Korean team respected each other and taught us a lot. After seeing how the Korean team worked, we said, ‘Let’s do this too!’ (Uzbek medical staff 3, 2017)

I learned from the Korean team how to communicate with patients. (Uzbek medical staff 1, 2017)

Usually, we only performed simple cases, but the Korean team performed difficult and complex ones. After the Korean team visited, our doctors and nurses worked harder. We began to open our books and study complex cases. This was a critical change in our team. (Uzbek medical staff 4, 2017)

Furthermore, the local medical staff became aware of the importance of teamwork while working with the Korean medical staff. Most participants stated that effective teamwork is one of the most important factors for more sustainable and successful surgery in the future.

The Korean team taught us ‘teamwork.’ They always emphasized, ‘Do not think that this is your patient if you conduct the surgery and this is my patient if I conduct the surgery. This is our patient whether the surgery is conducted by you or me.’ They also stressed that every member of the medical staff should make an effort to maintain the patients’ health irrespective of who conducts the surgery. Finally, we felt that we should work together for our patients’ health. (Uzbek medical staff 6, 2016)

It is common in Uzbekistan hospitals for doctors to usually make their own decisions. However, I think that the outcome of the surgery is good only if every team member does their own work in the team. No matter how well the thoracic surgeon does, a poor anesthesiologist cannot give patients good results. (Uzbek medical staff 1, 2017)

At the beginning of the program, the Uzbek medical staff felt that the obligation to take care of patients rested only on the medical staff who were involved in the surgery. During the ten year-long training, however, they realized that all medical staff were responsible for all patients’ health, and they had to closely collaborate for successful surgical outcomes.

### Theme 3: Life changes among patients, caregivers, and family members

Since most patients presented complex cases that were difficult for the Uzbek medical staff to successfully operate on, the patients and their caregivers, or family members, were satisfied with the results of the surgery conducted with collaboration between the Korean and Uzbek teams, and they experienced extensive life changes. Most of all, the surgery resulted in better health conditions, including higher physical performance for the children, which led to higher school attendance rates. Additionally, before surgery, the caregivers had to constrain their daily consumption because of high medical expenses involved in caring for heart disease, such as payments for transportation, medicine, and special meals for patients, and so on. After surgery, however, households could use their income for buying other necessary materials or save for the future.

After surgery, my child changed 100%. She does not catch cold or get sick anymore. She started walking and her growth increased gradually. (Caregiver 4, 2016)

Before the child had surgery, the family worried a lot. We were stressed out because we did not know if our child would survive. The atmosphere in the house was grim, and at that time I thought about leaving the family. (Caregiver 3, 2016)

The majority of caregivers were patients’ mothers. Prior to surgery, they could not participate in other economic activities or chores since they cared for their children. Some of them even felt guilty due to their child’s heart condition. After surgery, however, most caregivers began to lead their own life as a worker, housewife, or student. All these positive changes improved the quality of life of other family members as well.

Because my child was sick, I had to take care of my child all day. I could not do anything else or go anywhere. (Caregiver 1, 2016)

I wanted to continually study after having a child, but I could not. Now that my child is healthy, I want to start working or studying. (Caregiver 1, 2016)

### Theme 4: Challenges for next steps

Although the results showed positive effects in the capacity-building of the Uzbek medical staff and improvements in the quality of life of patients, several challenges remain as critical barriers to sustainably implement the program in the future.

The biggest obstacle was the lack of Korean medical volunteers. However, the composition of volunteers in every visit was also a challenging factor for the program’s sustainability.

Lack of Korean staff is always a challenging problem. While some of the staff travel there (Uzbek) as a business trip due to their hospital officially allowing them, based on the MOU [Memorandum of Understanding] with the JW LEE Center, the majority spend their personal vacation. In general, there are not enough nurses in each hospital, so it is hard for them to join this volunteer trip. (Korean medical staff 1, 2020)

It is difficult to recruit volunteers for every visit to Uzbekistan. However, I believe the greater concern is that the team’s composition is always changing because the members come from different hospitals. Since the medical styles and standards performed in each hospital are slightly different, the educational content which is taught to the Uzbek medical staff is not standardized. Although we had a workshop to share our common understandings, it was insufficient according to me. Given that the recruitment of medical staff will become more difficult in the future and its composition will change every time, it is urgent to prepare a standardized education manual for future intervention. (Korean medical staff 1, 2020)

Additionally, insufficient administrative and political support from the local hospital was also a limitation in developing the program.

While the Korean team taught us how to operate on complex cases, we could not continually conduct these surgeries after their visit as the hospital forbade the surgery of complex cases. The hospital was concerned that patients with significant difficulties would die because the hospital did not trust the surgical capabilities of the local medical staff. (Uzbek medical staff 4, 2016)

The hospital prohibited medical staff from conducting complex cases due to concerns about poor surgical outcomes. This prohibition might impede Uzbek surgical development by limiting the opportunities for conducting difficult surgery.

## Discussion

This study evaluated the effects of a ten-year-long cardiac surgical capacity-building program in Uzbekistan using mixed-methods. As a strength of this study, the mixed-methods approach allowed us to quantify the effects of the capacity-building program and to understand how team-based training was rooted in these effects. Through quantitative analysis, we showed that, as training progressed, both teams performed complex surgery cases and operated on patients with lower weights. The qualitative results also highlighted that team-based training, as a method of capacity-building, is one of the critical facilitators that improved the knowledge and skills of the local medical staff; and the developed capacity positively influenced the successful surgical outcomes and life changes among patients and their family members as well. Given the interview results, team-based training and activities reinforced the local team’s capacity [30].

It is noteworthy that the local staff were given the opportunity to perform complex cases with the Korean medical team and at times independently, under supervision of the Korean team, to develop their medical capacity [2,7,31]. According to the interviews, other foreign medical mission teams conducted surgery by themselves and the local staff were not given a chance to operate independently, or even under supervision, thereby creating difficulties in learning complex surgical skills. In contrast, this program empowered local staff by suggesting that they should lead complex surgical cases after adequate training under supervision of the visiting team. As previous studies have shown, close collaboration between the visiting team and the local team is an important way to effectively transfer medical knowledge and skills to the local medical staff [2,7,8,9]. Particularly, the Korean team educated the local team by matching their specialty, and this side-by-side training optimized the learning quality by not being constrained to a specific area and considering the local staff’s needs [7,32,33]. For instance, the two teams had multiple opportunities to discuss medical issues, such as future treatment plans, and care for patients in the ICU through formal or informal activities. They worked together at the hospital all week long, resulting in a trustworthy relationship.

The perceptions and behaviors of local medical staff toward patients changed as a result of the training team’s emphasis on a collective perspective regarding patients’ surgery outcomes. Before participating in this program, the local staff reported that they did not share the responsibility of a single patient and believed the individual who conducted surgery on the patient was the only one responsible. This mindset is common among physicians [7]. However, the local team learned to share responsibilities for both the processes and results of surgery after participating in the program, leading to a change in an important behavior that might facilitate positive results in health-professional training [7].

Our findings also provide four main challenges to the implementation of future programs in a more sustainable way. First, it is a critical challenge to recruit enough Korean medical volunteers in all specialties who can visit and conduct this program in LMICs. Although the recruitment of a sufficient number of skilled volunteers is essential to transfer medical skills to a local team [9,31,34], a large workload in Korean hospitals often hinders recruitment of the necessary volunteers at the right time. It will be possible to reduce the visiting team size if the local team’s capabilities are sufficiently improved [31,32]; but if not, then having an ample number of volunteers in diverse specialties is a critical precondition to successfully transmit medical skills to the local medical staff. Additionally, an online-based training program can be considered as an alternative to hands-on-training, and it can help to reduce the burden of recruiting human resources for conducting capacity-building programs [9,35].

Second, it is important to establish the team’s shared standards of medical operation for standardizing the training contents. Basically, the Korean surgical team composition was heterogeneous in medical background and experience because they were recruited from different hospitals. However, as our interviews and previous study have shown, it is important that all visiting members share a common understanding of medical operation before each visit in order to increase educational effectiveness by reducing these discrepancies [32]. To do so, standardized guidelines have to be established in the near future.

Third, although our program sustained for a decade to support the development of capacity and independence of the Uzbek medical team, a new strategy for the next decade should be established aiming to complete the independence of the Uzbek team [31]. As many studies have emphasized, establishment of a long-term strategy and commitment, of both the visiting team and the local receiving team, is one of the critical factors for the successful development of medical capacities in LMIC settings [1,5,31,36]. However, as other LMICs also experienced [2,31], insufficient support from the hospital administrators and funding to continue the program are important challenges for the near future [23]. Our ultimate goal should be to strengthen the team’s clinical skills and to communicate with important stakeholders.

Finally, the outcomes of our program should be periodically measured and evaluated by collecting data for program sustainability [2,5,32,33]. One of the limitations of our research was that we were not able to assess all surgical cases conducted at the institution because of time and cost constraints. In many cases, a well-established, well-managed database and quality assurance motivates all participants, as well as encourages achievement of the program’s objectives [33,37]. Data collection by the local medical team is also necessary to effectively monitor the interim outcomes themselves [32].

### Limitations

There are several limitations to this study. The first limitation is related to measurement issues for the outcomes; measurement and evaluation of the outcomes of pediatric cardiac surgery education programs are known to be difficult [32]. As a retrospective study without electronic medical records, the data were limited, and for some patients no information on outcomes was available. Follow-up visits also are limited as there are many barriers to accessing health care systems in LMICs. Therefore, we were not able to evaluate the post-operative patient outcomes in the long term. Hence, we focused on analysis of the process measures that were collected at the time of surgery, such as the complexity of cases and patient weights.

Furthermore, the qualitative data did not include all the Uzbek and Korean staff’s experiences or perceptions of the program due to the time limit. The interviews with caregivers were conducted only once after the surgery due to the costs of tracking patients and patients’ travel to the hospital [23]. Hence, accurate observation of changes in their quality of life before- and after intervention could not be explored.

Finally, this study was conducted with one surgical team in a single country. Therefore, the generalizability is limited, and the results should only be interpreted in the context of this study. While this case has limited generalizability, it can shed light on the planning and designing of other similar interventions.

## Conclusions

Our program evaluation research has added to previous studies on the advantages of team-based training, in this case, for improving pediatric cardiac surgical care in LMICs. The findings revealed the program’s multiple accomplishments and shortages, and areas for future improvement. The results of the current study will be applied to scale up the program in the future and to design a similar capacity-building program related to cardiac surgery in other developing-nation settings as well. While short-term, team-based training may not show immediate results, it can be an effective solution to increase and facilitate the local capacity for improving child health in relation to heart disease in LMICs.
